# Neuroprotective effects of antenatal magnesium sulfate on neurodevelopmental outcomes in very low birth weight preterm infants: A 2-year follow-up study

**DOI:** 10.1097/MD.0000000000043385

**Published:** 2025-07-18

**Authors:** Wai-Tim Jim, Jui-Hsing Chang, Hung-Yang Chang, Chun-Chih Peng, Chyong-Hsin Hsu

**Affiliations:** a Division of Neonatology, Department of Pediatrics, MacKay Children’s Hospital, Taipei City, Taiwan; b MacKay Medical College, New Taipei City, Taiwan; c MacKay Junior College of Medicine, Nursing and Management College, Taipei City, Taiwan; d National Taipei University of Nursing and Health Sciences, Taipei City, Taiwan.

**Keywords:** cerebral palsy, magnesium sulfate, neurodevelopmental impairment, periventricular leukomalacia, very low birth weight preterm infant

## Abstract

Antenatal magnesium sulfate (MgSO_4_) may provide neuroprotective benefits in preterm infants. This study examined the impact of antenatal MgSO_4_ on the neurodevelopmental outcomes of very low birth weight (VLBW) preterm infants at the corrected age of 2 years. This retrospective follow-up study included preterm infants with a birth weight ≤ 1500 g and gestational age ≤ 36 weeks who participated in a follow-up program. Antenatal MgSO_4_ was administered to treat maternal preeclampsia or for neuroprotection or tocolysis. Neurodevelopmental outcomes, including cerebral palsy (CP), neurodevelopmental impairment (NDI), and audiologic and visual assessments, were evaluated at a corrected age of 2 years. Infants exposed to MgSO_4_ were compared with unexposed controls. Among 328 VLBW infants (2007–2015), 133 were exposed to MgSO_4_, and 195 were not. Follow-up data were available for 93.3% of the infants. CP occurred in 6.0% of MgSO_4_ exposed infants versus 13.8% of controls (odds ratio: 0.39; 95% confidence interval: 0.18–0.91; *P* = .03). NDI was observed in 19.5% of exposed infants compared with 31.3% of controls (odds ratio: 0.53; 95% confidence interval: 0.32–0.90; *P* = .02). Multivariate logistic regression showed that low parental educational level (<college), birth weight (<1000 g), and periventricular leukomalacia were significantly associated with an increased rate of CP. Similarly, independent factors such as preeclampsia, low socioeconomic status, birth weight < 1000 g, male sex, and periventricular leukomalacia were significantly associated with an increased risk of NDI. Antenatal MgSO_4_ administration in pregnant women may have neuroprotective effects in VLBW preterm infants, reducing the risk of CP and NDI at the corrected age of 2 years.

## 1. Introduction

Although the survival rate of very low birth weight (VLBW, birth weight < 1500 g) preterm infants has improved owing to advanced perinatal care, cerebral palsy (CP) and neurodevelopmental impairment (NDI) remain the main neurodevelopmental complications associated with prematurity.^[1–6]^ CP and NDI in preterm infants may cause profound medical, emotional, and economic consequences during their childhood.^[6–9]^ The incidence of CP and NDI in VLBW infants was approximately 12.7% and 29.5%, respectively.^[6,9]^ Therefore, early recognition and intervention of CP and NDI in VLBW infants was one of the major recommendations by the American Academy of Pediatrics.^[10]^

Based on a study by Steer and Petrie,^[11]^ magnesium sulfate (MgSO_4_) was first used as an effective tocolytic agent to arrest labor. MgSO_4_ was then used as an anticonvulsant agent in preeclampsia and subsequently as a tocolytic and neuroprotective agent.^[12,13]^ During the last 2 decades, new data from observational studies and randomized controlled trials suggested that antenatal MgSO_4_ was associated with a reduced risk of CP in VLBW infants.^[14–18]^ Conversely, Mittendorf et al^[19]^ found that the use of antenatal MgSO_4_ was associated with worse perinatal outcomes in a dose-dependent fashion. Furthermore, Bekmez et al recently observed a potential association between antenatal MgSO_4_ exposure and feeding intolerance (FI) in VLBW infants.^[20]^ Hence, this study examined the database regarding the neuroprotective effects of MgSO_4_ on neurodevelopmental outcomes in VLBW preterm infants at the corrected age of 2 years.

## 2. Methods

Singleton, twin, or triplet VLBW preterm infants in this study were born at Mackay Memorial Hospital between January 2007 and December 2013. The follow-up period was extended to December 2015 to ensure longitudinal data collection. The estimated gestational age (GA) was derived from menstrual history and the results of the earliest sonography examination. The GA in the study ranged from 23 weeks to 36 weeks. The clinical characteristics of all participants, including GA, birth body weight, sex ratio, multiple gestations, Neonatal Therapeutic Intervention Scoring System, Apgar scores at 1 and 5 minutes after birth, initial pH, intubation or not, inotropic agent use, and clinical diagnosis of sepsis, respiratory distress syndrome, necrotizing enterocolitis (NEC, stage II or III), FI (presence of gastric residual volume > 50% of the previous feeding and abdominal distension or vomiting),^[21]^ intraventricular hemorrhage (IVH), and periventricular leukomalacia (PVL), were reviewed and obtained after the infants’ admission and before their discharge from neonatal units.

The preterm follow-up program further examined the neurological outcomes of preterm infants, including CP, NDI, and mental development index (MDI) and psychomotor development index (PDI) on the Bayley Scales of Infant Development (BSID). Visual and hearing functions of infants were assessed by neonatologists, pediatric neurologists, and clinical psychologists after discharge.

Infants with major congenital abnormalities, chromosomal anomalies, congenital infections, brain malformations, a clinical diagnosis of asphyxia/hypoxic-ischemia encephalopathy, or metabolic diseases were excluded from this study. Infants without BSID-II scores, BSID-III scores, or complete follow-up visits were excluded.

The obstetric records of the mothers were also reviewed. The clinical characteristics of the infants’ mothers, including age, multiparity, multiple pregnancies, preeclampsia with or without tocolysis, use of antenatal steroids, fetal distress, delivery mode, socioeconomic status, and parental education level, were also obtained. The socioeconomic levels were classified as low and high if the annual median household income was ≤USD 2024.40 and ≥USD 4050.00, respectively.^[22]^ Antenatal MgSO_4_ was administered for maternal preeclampsia, neuroprotection, or tocolysis. The administration method was based on that of Crowther et al^[12,16]^ which involved a 4 g (16 mmol) intravenous bolus of MgSO_4_ followed by a maintenance infusion of 1 g/h. The vital signs of the patients were monitored throughout treatment. The control infants did not receive any antenatal MgSO_4_.

### 2.1. Neurodevelopmental assessment and outcomes

Two neonatologists and a certified research nurse collected the data at scheduled follow-up visits when the infants reached 6, 12, and 24 months corrected age. CP is a group of disorders involving abnormal movement and posture that lead to activity limitation. These disorders result from nonprogressive disturbances in the infant brain.^[23]^ All cases and control infants underwent cranial sonography within the first week of life and during hospitalization. Additional scans were performed after discharge. IVH was defined based on the Papile Grading System.^[24]^ PVL was diagnosed using ultrasound or MRI.

The second and third editions of the BSID (BSID-II and BSID-III, respectively) were used to assess neurodevelopmental outcomes at a corrected age of 2 years (corrected for GA at birth using the mother’s expected date of confinement). Trained clinical psychologists performed the assessments. BSID-II, MDI, and PDI scores were collected. An MDI < 70 or a PDI < 70 of the BSID-II was used, representing 2 standard deviations below the mean and corresponding to a significant developmental delay. Cognitive, language, and motor scores of the BSID-III were also collected, and a combined Bayley-III score < 85 was defined as moderate-to-severe neurodevelopmental delay, equivalent to an MDI < 70 on the BSID-II. Auditory evoked potentials were examined in all patients at corrected ages of 12 and 24 months. Furthermore, visual evoked potentials were performed only in infants with suspected clinical visual impairment.

NDI was defined as any of the following: MDI < 70, PDI < 70, BSID-III < 85, CP, visual impairment, or hearing impairment.^[25]^

This study was approved by the Institutional Review Board of MacKay Memorial Hospital (IRB number: 23MMHIS327e). All patient records and information were anonymized and de-identified before analysis, and all schemes in this study adhered to the tenets of the Declaration of Helsinki.

### 2.2. Statistical analysis

Maternal and infant categorical variables were collected and compared using Fisher exact test or the chi-square test. All continuous parametric data were analyzed using two-tailed unpaired *t* tests for between-group comparisons. Univariate analysis was used to estimate crude odds ratios (ORs) and 95% confidence intervals (CIs). The CIs of the variables with the value 1 were not included in the multivariate analyses. To avoid missing potentially significant factors, we entered each variable with a *P* < .1 by univariate analysis into the multivariate logistic regression model. Multivariate logistic regression analyses were performed to calculate the adjusted ORs and 95% CIs to identify risk factors that were significantly associated with the outcomes of CP and NDI, respectively. All statistical analyses were performed using SPSS Statistics for Windows (Version 27.0). Statistical significance was set at *P* < .05. All *P*-values reported were two-tailed.

## 3. Results

### 3.1. Clinical characteristics of mothers

Among 328 VLBW infants enrolled in the study between January 2007 and December 2015, 133 (40.5%) had antenatal MgSO_4_ exposure (MgSO_4_ group), and 195 (59.5%) had no antenatal MgSO_4_ exposure (control group). Follow-up was achieved in 93.3% of the infants, with 22 excluded because their parents did not participate in the follow-up program. The age of mothers was greater in the MgSO_4_ group than mothers in the control group (33.3 ± 4.3 vs 31.6 ± 5.2 years; odds ratio [OR]: 1.08; 95% CI: 1.03–1.13; *P* < .01), particularly in maternal age < 30 years (28.0 ± 1.6 vs 26.7 ± 3.4 years; OR: 1.21; 95% CI: 1.01–1.45; *P* = .04). There were higher incidences of preeclampsia (54.9 vs 4.1%; OR: 2.13; 95% CI: 1.76–2.57; *P* = .001), antenatal steroid use (85 vs 72.8%; OR: 1.18; 95% CI: 1.14–2.88; *P* = .01), fetal distress (39.1 vs 21%; OR: 0.53; 95% CI: 0.38–0.76; *P* < .01), and cesarean section of delivery mode (83.5 vs 64.6%; OR: 2.76; 95% CI: 1.61–4.76; *P* < .01) in the MgSO_4_ group. Other characteristics such as multiparity, multiple pregnancies, parental education level, and socioeconomic status showed no significant differences between the MgSO_4_ and control groups (Table 1).

**Table 1:** Univariate analysis of maternal clinical characteristics between MgSO_4_ and control groups during treatment and delivery.

	MgSO_4_ (N = 133)	Control (N = 195)	OR (95% CI)	*P* value
Maternal age (years)*	33.3 ± 4.3	31.6 ± 5.2	1.08 (1.03–1.13)	<.01
≤30	28.0 ± 1.6	26.7 ± 3.4	1.21 (1.01–1.45)	.04
31–39	34.2 ± 2.6	34.4 ± 2.4	0.97 (0.87–1.09)	.65
≥40	41.7 ± 2.2	42.4 ± 2.3	0.86 (0.55–1.34)	.50
Multiparity	87 (65.4)	122 (62.6)	0.96 (0.81–1.13)	.64
Multiple pregnancies	42 (31.6)	47 (24.1)	0.76 (0.54–1.09)	.16
Preeclampsia	73 (54.9)	8 (4.1)	2.13 (1.76–2.57)	.001
Antenatal steroids	113 (85)	142 (72.8)	1.18 (1.14–2.88)	.01
Fetal distress	52 (39.1)	41 (21.0)	0.53 (0.38–0.76)	<.01
Delivery mode				
NSD	22 (16.5)	69 (35.4)	2.76 (1.61–4.76)	<.01
Cesarean section	111 (83.5)	125 (64.6)		
Education level (no.)	127	186		
<College	48 (37.8)	72 (38.7)	0.96 (0.61–1.53)	.87
>College	79 (62.2).	114 (61.3)		
Socioeconomic status (no.)†	128	186		
Low	54 (42.2)	95 (51.1)	1.25 (0.98–1.59)	.61
High	74 (57.8)	91 (48.9)		

CI = confidence interval, MgSO_4_ = magnesium sulfate, NSD = normal spontaneous delivery, OR = odds ratio.

* Continuous data expressed as mean ± SD, categorical data expressed as no. of case (%).

† The socioeconomic levels were classified as low and high if the annual median household income was ≤USD 2024.40 and ≥USD 4050.00, respectively.

### 3.2. Clinical characteristics of VLBW infants

Similarly, GA of infants (29.6 ± 2.7 vs 28.9 ± 2.9 weeks; OR: 1.09; 95% CI: 1.01–1.18; *P* = .03), particularly in the GA ≤ 28 weeks (26.9 ± 1.3 vs 26.1 ± 1.5 weeks; OR: 1.45; 95% CI: 1.12–1.89; *P* < .01), were greater in the MgSO_4_ group than in the infants in the control group. Other variables including birth weight, sex ratio, multiple gestations (43 pairs of twins and one set of triplets), Apgar score in 1st and 5th min after birth, first pH value of blood gas, use of intubation, inotropic agent use, sepsis, NEC, FI, respiratory distress syndrome, and IVH showed no significant differences between the groups. However, the incidence of PVL was lower in the MgSO_4_ group than in the control group (1.5% vs 7.2%; OR: 0.19; 95% CI: 0.04–0.88; *P* = .02) (Table 2).

**Table 2:** Univariate analysis of clinical characteristics of VLBW infants between MgSO_4_ and control groups before hospital discharge.

	MgSO_4_ (N = 133)	Control (N = 195)	OR (95% CI)	*P* value
Gestational age (weeks)*	29.6 ± 2.7	28.9 ± 2.9	1.09 (1.01–1.18)	.03
≤28	26.9 ± 1.3	26.1 ± 1.5	1.45 (1.12–1.89)	<.01
>28	31.3 ± 1.9	30.9 ± 1.9	1.09 (0.94–1.27)	.27
Birth body weight (g)*	1141.8 ± 258.7	1112.9 ± 273.8	1.00 (1.00–1.00)	.33
<1000	797.2 ± 110.4	775.8 ± 126.9	1.00 (0.99–1.00)	.37
1000–1500	1290.1 ± 130.4	1277.6 ± 144.1	1.00 (0.99–1.00)	.51
Sex				
Male	70 (52.6)	90 (46.2)	1.14 (0.91–1.42)	.26
Female	63 (47.7)	105 (53.8)	0.88 (0.70–1.09)	
Multiple gestations	42 (31.6%)	47 (24.1%)	1.45 (0.89–2.38)	.14
1st minute Apgar score ≤ 6	65 (48.9)	86 (44.1)	0.90 (0.71–1.14)	.43
5th minute Apgar score ≤ 6	18 (13.5)	17 (8.7)	0.64 (0.35–1.20)	.20
First pH <7.0	1 (0.8)	6 (3.1)	3.86 (0.47–31.65)	.25
Intubation	80 (60.2)	119 (61.0)	1.01 (0.85–1.20)	.91
Inotropic agents use	33 (24.8)	52 (26.7)	1.08 (0.74–1.57)	.78
Sepsis	24 (18.0)	37 (18.9)	1.05 (0.66–1.67)	.89
NEC stage (II or III)	1 (0.8)	1 (0.5)	NA	NA
Feeding intolerance	17 (12.8)	23 (11.8)	0.92 (0.51–1.66)	.79
RDS	96 (72.1)	134 (68.7)	0.96 (0.83–1.10)	.62
IVH	19 (14.3)	28 (14.4)	1.01 (0.59–1.72)	1.00
Mild IVH (grade 1–2)	16 (12.0)	25 (12.8)	1.06 (0.54–2.08)	.85
Severe IVH (grade 3–4)	3 (2.3)	3 (1.5)	0.68 (0.13–3.44)	.64
PVL	2 (1.5)	14 (7.2)	0.19 (0.04–0.88)	.02

CI = confidence interval, CP = cerebral palsy, IUGR = intrauterine growth retardation, IVH = intraventricular hemorrhage, MgSO_4_ = magnesium sulfate, NA = not available, NEC = necrotizing enterocolitis, OR = odds ratio, PVL = periventricular leukomalacia, RDS = respiratory distress syndrome, VLBW = very low birth weight.

* Continuous data expressed as mean ± SD, categorical data expressed as no. of case (%).

### 3.3. Neurodevelopmental outcomes

This study showed that 6% (8 of 133) of the infants with antenatal MgSO_4_ and 13.8% (27 of 195) of the controls had CP (adjusted odds ratio [aOR]: 0.39; 95% CI: 0.17–0.91; *P* = .03), and 19.5% (26/133) of the infants with MgSO_4_ treatment and 31.3% (61/195) in the controls had NDI (aOR: 0.52; 95% CI: 0.30–0.90; *P* = .02). There was a significantly lower rate of MDI < 70 (5.0% vs 15.6%; aOR: 0.27; 95% CI: 0.11–0.68; *P* = .01) and the PDI < 70 (16.8% vs 27.3%; aOR: 0.50; 95% CI: 0.27–0.93; *P* = .02) in the MgSO_4_ group than in the control group. The neurodevelopmental assessment data, including the results of hearing and visual function tests, and BSID-II and BSID-III for neurodevelopmental outcomes are shown in Table 3. The decreased frequency of CP, NDI, MDI < 70, and PDI < 70 in infants treated with antenatal MgSO_4_ suggests that there might be neuroprotective effects.

**Table 3:** Univariate and multivariate logistic regression analysis of neurodevelopment outcome between MgSO_4_ and control groups at corrected age of 2 years.

Variables	MgSO_4_ no. (N = 133)	Control no. (N = 195)	Unadjusted		Adjusted	
			OR (95% CI)	*P* value	aOR (95% CI)	*P* value
Abnormal AEP	9 (6.77)	17 (8.72)	0.76 (0.33–1.76)	.52	0.84 (0.35–1.99)	.69
Abnormal VEP	4/9 (44.4)	12/20 (60.0)	0.53 (0.11–2.61)	.68	NA	NA
Cerebral palsy	8 (6.0)	27 (13.8)	0.39 (0.18–0.91)	.03	0.39 (0.17–0.91)	.03
NDI	26 (19.5)	61 (31.3)	0.53 (0.32–0.90)	.02	0.52 (0.30–0.90)	.02
Bayley Scales of Infant Development-II, BSID-II						
MDI < 70	6/119 (5.0)	25/161 (15.6)	0.29 (0.11–0.72)	.01	0.27 (0.11–0.68)	.01
PDI < 70	20/119 (16.8)	44/161 (27.3)	0.54 (0.29–0.97)	.03	0.50 (0.27–0.93)	.03
Bayley Scales of Infant Development-III, BSID-III						
Cognitive score < 85	3/7 (42.8)	2/19 (10.5)	NA	.83	NA	NA
Language score < 85	2/7 (28.5)	6/19 (31.6)	NA	.33	NA	NA
Motor score < 85	3/7 (42.8)	10/19 (52.6)	NA	.31	NA	NA

Categorical data expressed as no. of case (%); NA: not available.

AEP = auditory evoked potentials, BSID = Bayley Scales of Infant Development, CI = confidence interval, CP = cerebral palsy, EEG = electroencephalogram, MDI = mental development index, MgSO_4_ = magnesium sulfate, NDI = neurodevelopmental impairment, OR = odds ratio, PDI = psychomotor development index, VEP = visual evoked potentials.

Additional analysis was performed to elucidate the independent factors for CP and NDI survival from hospital discharge to follow-up visits at a corrected age of 2 years.

Significant maternal and neonatal factors, including MgSO_4_, maternal age, multiple pregnancies, fetal distress, preeclampsia, antenatal steroid use, delivery mode, parental educational level and socioeconomic status, GA, birth weight, and sex, were selected for further multivariate logistic regression analysis following the univariate logistic regression model.

### 3.4. Risk factors of CP and NDI

Multivariate logistic regression analysis of risk factors revealed that a low parental educational level (<college) (aOR: 2.40; 95% CI: 1.08–5.34; *P* = .03), birth weight (<1000 g) (aOR: 2.34; 95% CI: 1.04–5.29; *P* = .04), and PVL (aOR: 26.76; 95% CI: 7.99–89.57; *P* < .001) were significantly associated with an increased rate of CP.

Similarly, independent factors such as preeclampsia (aOR: 2.43; 95% CI: 1.15–5.12; *P* = .02), low socioeconomic status (aOR: 3.28; 95% CI: 1.80–5.98; *P* < .01), birth weight < 1000 g (aOR: 2.81; 95% CI: 1.59–4.95; *P* < .01), male sex (aOR: 1.82; 95% CI: 1.04–3.19; *P* = .04), and PVL (aOR: 8.45; 95% CI: 2.45–29.10; *P* < .01) were significantly associated with an increased risk of NDI.

## 4. Discussion

This study showed that infants with antenatal MgSO_4_ had a lower rate of CP and NDI than the control infants. Magnesium can block the N-methyl-D-aspartate receptor, which modulates inflammation, stabilizes intracranial tone, reduces fluctuations in cerebral blood flow, and prevents calcium-mediated intracellular damage in humans.^[26,27]^ Nizar et al reported that MgSO_4_ might prevent preterm brain injury by inhibiting inflammation, apoptosis, and oxidative stress.^[28]^

Several professional societies have published practice guidelines on the use of MgSO_4_ for fetal neuroprotection in cases of preterm birth, consistently recommending its use.^[12,29,30]^ The International Federation of Gynecology and Obstetrics and the Society of Obstetricians and Gynaecologists of Canada Clinical Practice Guideline recommend administering a loading dose of 4 g of MgSO_4_ > 20 to 30 minutes, with or without a maintenance dose of 1 g/hour until delivery, for pregnant women at <34 weeks of gestation with imminent preterm birth. Additionally, the American College of Obstetricians and Gynecologists reaffirmed in 2022 that MgSO_4_ administered before anticipated early preterm birth (<32 weeks of gestation) reduces the risk of CP in surviving infants. The guideline recommends an initial bolus dose of 4 to 6 g of MgSO_4_, with the option to treat women with active preterm labor with or without a maintenance dose.

The optimal regimen that achieves maximum effectiveness with minimal adverse effects for the fetus and mother is yet to be determined. However, the neuroprotective role of antenatal MgSO_4_ in preterm infants was well established.^[18,31]^ McNamara et al^[32]^ examined various MgSO_4_ regimens administered to mothers to prevent preterm labor. However, evidence regarding the optimal dose (including loading and maintenance doses), therapy duration, administration timing, and the role of repeat dosing in ensuring efficacy and safety for mothers and their children remains insufficient.

Mittendorf et al^[19]^ reported adverse outcomes associated with antenatal MgSO_4_ use in preterm infants. In addition, infants exposed to antenatal MgSO_4_ might exhibit temporary flaccidity or respiratory depression.^[33]^ Recent studies have also suggested a potential association between antenatal neuroprotective MgSO_4_ use and the development of NEC or FI in VLBW infants.^[20,34]^ Therefore, informing mothers about the relative advantages and disadvantages of antenatal MgSO_4_ use is essential.

Conversely, Weisz et al^[35]^ found that the intrapartum use of MgSO_4_ for fetal neuroprotection was not associated with increased intensive delivery room resuscitation. Moreover, a systematic review by Shepherd et al did not find clear associations between antenatal MgSO_4_ for beneficial indications and adverse neonatal outcomes.^[36]^ Consistent with these findings, this study did not observe clinical adverse effects in infants during hospitalization following antenatal MgSO_4_ administration.

Shepherd et al,^[18]^ in the Cochrane Database, reported that antenatal administration of MgSO_4_ for women at risk of preterm birth reduced the risk of CP and the combined outcome of death or CP in children at a corrected age of 2 years. Additionally, it is likely to reduce the incidence of severe IVH in infants. The findings of this study suggest that antenatal MgSO_4_ offers neuroprotection for VLBW preterm infants.

Preterm infants, particularly those born to high-risk pregnant women, are vulnerable to CP and NDI. The incidence of CP in this study was 10.7%, which falls within the range reported in previous large-scale studies (5.9–14%).^[37–39]^ Risk factors for CP in preterm infants include maternal age, maternal infection, parental education level, socioeconomic status, male sex, low birth weight, hyperbilirubinemia, hypoglycemia, and cystic PVL.^[4,9,40]^ In this study, the risk factors associated with CP at a corrected age of 2 years included low parental education level (< college), birth weight < 1000 g, and PVL. These findings were consistent with those of previous studies.

Approximately 26.5% of infants with NDI at a corrected age of 2 years were identified in the study, falling within the 7% to 37% range reported in previous studies on infants with extremely low birth weight or GA.^[5,41]^ Risk factors for NDI in preterm infants include maternal education level (lower than high school), GA of <34 weeks, high-grade IVH (grades 3–4), PVL, bronchopulmonary dysplasia, sepsis, non-English-speaking households, CP, and lower socioeconomic status.^[4,8,40]^ The findings indicated that preeclampsia, low socioeconomic status, birth weight of <1000 g, male sex, and PVL were significantly associated with an increased risk of NDI, which aligns with previous reports.

This study has several limitations. First, this was not a randomized prospective follow-up study. Moreover, the sample size was small, and the follow-up duration should have been longer. Although pediatric neurologists and rehabilitation physicians diagnosed CP in preterm infants during follow-up visits, we did not further investigate the subtypes of CP and the severity of NDI. Unsurprisingly, multivariate logistic regression analysis did not reveal a significant association between antenatal MgSO_4_ and outcomes such as CP and NDI, as factors such as birth weight (<1000 g) and PVL predominantly influenced the results. Furthermore, the magnesium levels in the blood were not measured in any patient. Despite these limitations, we recommend conducting a prospective, randomized, controlled, long-term cohort study to explore this issue further.

In summary, antenatal MgSO_4_ plays a crucial role in neuroprotection during high-risk pregnancies, particularly in reducing the risk of CP and NDI in preterm infants. Recognizing parental and neonatal risk factors enables targeted care strategies, early referrals, and timely interventions to improve neurodevelopmental outcomes in VLBW preterm infants at a corrected age of 2 years.

## Acknowledgments

The authors thank all the parents and infants who participated in this study. We also thank Ray Chen, MD, and Chia-Ming Pai, MD, for writing the first draft and assisting with initial data collection; S.L. Chim and N.Y. Yim, MSc, for their critical revision of the manuscript and support with statistical analysis.

## Author contributions

**Conceptualization:** Wai-Tim Jim, Jui-Hsing Chang.

**Data curation:** Wai-Tim Jim.

**Formal analysis:** Jui-Hsing Chang, Hung-Yang Chang.

**Investigation:** Wai-Tim Jim, Jui-Hsing Chang, Hung-Yang Chang, Chun-Chih Peng.

**Methodology:** Jui-Hsing Chang, Hung-Yang Chang.

**Supervision:** Chun-Chih Peng, Chyong-Hsin Hsu.

**Writing – original draft:** Wai-Tim Jim, Jui-Hsing Chang.

**Writing – review & editing:** Wai-Tim Jim, Jui-Hsing Chang.
